# Hemophagocytic lymphohistiocytosis resulting from a cytokine storm triggered by septicemia in a child with chronic granuloma disease: a case report and literature review

**DOI:** 10.1186/s12887-020-1996-3

**Published:** 2020-03-03

**Authors:** Ang Wei, Honghao Ma, Liping Zhang, Zhigang Li, Qing Zhang, Dong Wang, Li Zhang, Hongyun Lian, Rui Zhang, Tianyou Wang

**Affiliations:** 1 Beijing Key Laboratory of Pediatric Hematology Oncology; National Key Discipline of Pediatrics (Capital Medical University); Key Laboratory of Major Diseases in Children, Ministry of Education; Hematology Oncology Center, Beijing Children’s Hospital, Capital Medical University, National Center for Children’s Health, Beijing, 100045 China; 2 Hematology and Oncology Laboratory, Beijing Pediatric Research Institute, National Center for Children’s Health, Beijing Key Laboratory of Pediatric Hematology Oncology, Key Laboratory of Major Diseases in Children, Ministry of Education, National Key Discipline of Pediatrics, Beijing Children’s Hospital Affiliated to Capital Medical University, Beijing, 100045 China; 3 Department of Hematology and Oncology, Beijing Children’s Hospital, Capital Medical University, Nanlishi Road No. 56, Xicheng District Beijing, 100045 P.R. China

**Keywords:** Chronic granuloma disease, Hemophagocytic Lymphohistiocytosis, Pathogenesis, Case report, IVIG

## Abstract

**Background:**

Hemophagocytic lymphohistiocytosis (HLH) is a rare potentially fatal illness characterized by impaired natural killer and cytotoxic T cell function. Chronic granulomatous disease (CGD) is an inherited immune deficiency caused by a defect in the nicotinamide adenine dinucleotide phosphate (NADPH) oxidase complex. CGD patients display an increased susceptibility to infection with bacteria and fungi. Repeated infections lead to an increased risk for developing HLH. The case of CGD with repeated Salmonella septicemia complicated with HLH is very rare, and the CGD mutation identified has not been reported.

**Case presentation:**

A 3-year-old boy was admitted to our hospital for fever, hepatosplenomegaly and pancytopenia. According to the clinical manifestations and laboratory results, hemophagocytic lymphohistiocytosis (HLH) was diagnosed. Blood and bone marrow culture confirmed septicemia due to Salmonella Typhimurium. On the basis of antiinfection treatment, methylprednisolone was used to control HLH. After treatment, the clinical symptoms and laboratory results improved. Gene analysis showed a novel hemizygous CYBB gene mutation: c.302A > G (p.H101P). Combined with a past history of recurrent infection, the child was diagnosed with HLH secondary to CGD triggered by septicemia.

**Conclusions:**

In case of a known (or highly suspected) CGD with a documented infection, clinical or biological features of HLH should encourage the physician to make possible to confirm or not the HLH. Therefore, to initiate the adequate treatment in association with anti-infective therapy.

## Background

Hemophagocytic lymphohistiocytosis (HLH) is a rare potentially fatal illness characterized by impaired natural killer (NK) and cytotoxic T cell function. Patients present with hemophagocytosis, cytopenia, and multiorgan failure [1]. HLH can be categorized into two distinct forms: primary or familial HLH (FHL) and secondary HLH. Primary HLH occurs mostly in the presence of an underlying predisposing genetic defect in immune function, and secondary HLH may result from malignant, infectious, or autoinflammatory diseases [2, 3].

Chronic granulomatous disease (CGD) is an inherited immune deficiency caused by a defect in the nicotinamide adenine dinucleotide phosphate (NADPH) oxidase complex. Most cases are X linked and result from defects in the CYBB gene, while others have an autosomal recessive pattern [4]. CGD patients display an increased susceptibility to infection with bacteria and fungi. Repeated infections result in an increased risk for developing HLH. Infection-associated hemophagocytic syndrome (IASH) with CGD has been observed in eight children and two adults [5–9]. We report a case of CGD with repeated Salmonella septicemia complicated with HLH, and the CGD mutation identified has not been reported.

## Case presentation

In April 2019, a 3-year-old boy was referred to our hospital with a 4-week history of recurrent fever (39.5 °C), progressive hepatosplenomegaly and pancytopenia. From birth to presentation, the boy displayed repeated pneumonia 3 times, each lasting approximately 2 weeks, which improved on antibiotics. The boy was hospitalized at 1 year of age for a perianal abscess and at 1.5 years of age for septicemia caused by Salmonella Typhimurium. There was no family history of immunodeficiencies, autoimmune diseases or early childhood deaths.

**Fig. 1:**
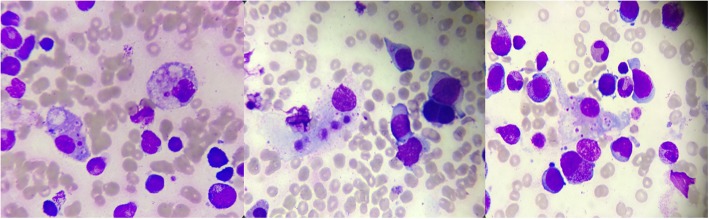
Bone marrow smear showing the hemophagocytic phenomenon

His initial physical examination showed normal blood pressure and high body temperature (38.5 °C). Remarkable lymphadenopathy was observed throughout the bilateral neck, with soft quality and clear boundaries. Laboratory tests revealed pancytopenia. Elevated levels of C-reactive protein, lactic dehydrogenase, serum ferritin, sCD25, triglyceride (5.99 mmol/L), IFN-γ, IL-6, IL-18, IL-1β, TNF-α and IL-10 were also observed. The fibrinogen level and NK cell activity were below the normal range (Table 1). A bone marrow aspirate showed hemophagocytosis (Fig. 1). Blood and bone marrow culture confirmed the presence of Salmonella Typhimurium. Serological tests for CMV, EBV, HSV and other pathogens were all negative results. Biochemical tests, protein content and cell count of the cerebrospinal fluid were within the normal range. An abdominal ultrasound scan revealed massive hepatosplenomegaly accompanied by a large amount of calcification. His family history was unremarkable.

**Table 1:** Cliniacal characters

Variable	Reference Range	D1	D10
Temp (°C)	–	39.5	36.3
Lver	–	5 cm under rib	Normal
Spleen	–	8 cm under rib	Normal
Hemoglobin (g/L)	110–190	62.1	106
Leukocyte (cells/L)	4–10 × 10^9	1.2	4.8
Platelet count (cells/L)	100–400 × 10^9	19	189
C-reaction protein (mg/dl)	0–8	76	12
Fibrinogen (g/L)	2–4	1.42	1.94
Lactic dehydrogenase (g/L)	110–295	1119	249
Triglyceride (mmol/L)	0–2	5.88	3.89
Serum ferritin (ng/ml)	10–120	4758	697
sCD25 (pg/ml)	0–6400	37,202	567
IFN-γ (pg/ml)	1.60–17.30	964.37	0.9
IL-6 (pg/ml)	1.7–16.6	524.13	3.06
IL-18 (pg/ml)	0–6.5	936.8	–
IL-1β (pg/ml)	0–0.7	11.5	–
TNF-α (pg/ml)	0.1–5.2	11.8	1.06
IL-10 (pg/ml)	2.6–4.9	26.08	2.86
NK cell activity (%)	≥15.11	14.18	–

Based on the presence of the 8 HLH-2004 diagnosis criteria of fever, splenomegaly, pancytopenia, hypertriglyceridemia, hypofibrinogenemia, histological evidence of hemophagocytosis, hyperferritinemia, elevated levels of sCD25 and low NK cell activity, a diagnosis of HLH was made. Due to the obvious increase in IFN, IL-10 and sCD25 levels, we considered the condition of HLH to be serious. Methylprednisolone (10 mg/kg/d) was given for 3 days, then at 5 mg/kg/d for 3 days, at 2 mg/kg/d for 3 days, and finally tapered to 1 mg/kg/d oral administration. However, due to the unknown etiology and significantly high level of CRP, serious infection could not be ruled out. Therefore, we did not add VP16 for chemotherapy. On day 3, septicemia was diagnosed based on the results of blood and bone marrow culture, and meropenem was given according to the results of a drug sensitivity test.

After 1 week of antiinfection and methylprednisolone treatment, the child’s situation improved. His hemoglobin increased to 106 g/L, his WBC count improved to 4.8 × 10^9^ cells/L, and his platelet count was 189 × 10^9^ cells/L. The levels of serum ferritin (697 ng/ml), sCD25 (567 pg/ml), IFN-γ (0.9 pg/ml) and IL-6 (3.06 pg/ml) decreased, and the fibrinogen level (1.56 g/l) increased (Table 1). Two weeks after treatment, mutation analysis revealed that the patient carried a hemizygous mutation in the CYBB gene (c.302A > G (p. H101P)) encoding the gp91phox protein. The mutation rate of this locus is low in the normal population, and it has not yet been reported in the relevant literature. CGD was diagnosed based on repeated bacterial infection and genetic results. Therefore, the patient had IASH secondary to CGD triggered by Salmonella Typhimurium septicemia.

## Discussion and conclusions

In this case, fever, hepatosplenomegaly and pancytopenia were the main manifestations, combined with Salmonella Typhimurium septicemia, which met the diagnostic criteria of IASH. Gene analysis showed a novel hemizygous CYBB gene mutation: c.302A > G (p.H101P). Combined with a past history of recurrent infection, the child was diagnosed with HLH secondary to CGD triggered by septicemia.

CGD patients are considered to have a defect in the respiratory burst of phagocytes but not in their activation. When septicemia occurs in children, the cells cannot destroy the bacteria, but their proinflammatory cytokine production, such as that of IL-1, IL-10 and TNF-α, is increased, which additionally causes a cytokine storm, leading to a massive activation of macrophages and lymphocytes and making the patient prone to developing HLH [7]. Jackson et al. [10] found that in a mouse model of CGD, the lack of the NADPH oxidase complex in T lymphocytes decreased the receptor number, which increased the release of Th1 cytokines in mice. Th1 cytokines such as IFN-γ can induce granulocyte-macrophage activation and eventually induce HLH [11]. Therefore, an inflammatory cytokine storm secondary to CGD infection was the main cause of HLH in this child.

For the diagnosis of CGD associated with HLH, it was usually believed that the blood cell reduction, ferritin increase, splenomegaly and bone marrow showing hemophagocytosis in CGD patients are manifestations of septicemia caused by severe infection, while the diagnosis of HLH is ignored. However, unlike in septicemia caused by severe infection, children with HLH show a decrease in NK cell activity and a significant increase in IFN-γ and IL-10 levels [12]. Bacterial infections such as septicemia cause mainly an increase in IL-6 [13]. The increased cytokines in this child were mainly HLH-specific IFN-γ and IL-10, while septicemia-related IL-6 was only slightly increased, indicating that the fever, hepatosplenomegaly and hematopenia were related mainly to HLH. On day 3, we added methylprednisolone to control the cytokine storm, and the clinical and laboratory parameters of HLH improved quickly after treatment with the immunosuppressant. Early identification, diagnosis and treatment are of great significance for the prognosis of patients.

CGD is an immunodeficiency disease, while HLH is caused by excessive inflammation in the body. Therefore, the treatment of CGD associated with HLH should be very cautious. Different from the traditional HLH-94/04 regimen, CGD associated with HLH should choose drugs with weaker immunosuppressive effects, such as intravenous immunoglobulin (IVIG) or corticosteroids. Drugs with strong immunosuppressive effects, such as cyclosporine and etoposide, should be avoided. There are some reports noting that although IVIG is not the first-line treatment for patients with CGD, it appears to be effective because of its powerful immunomodulatory effect and safety profile [7]. Parekh reported 3 patients with CGD associated with HLH. After administering steroids, IVIG and antibiotics, the clinical and laboratory parameters were significantly improved [5]. Álvarez-Cardona also reported two cases of CGD complicated by MAS that were successfully treated with IVIG [7]. For such patients, using antibiotic drugs alone cannot achieve a good response [9]. Our patient was not diagnosed with CGD at admission, but he was young and had repeated infections after birth, suggesting that he might have a congenital immune deficiency. Therefore, although this child was diagnosed with HLH after admission, we did not give him intensive chemotherapy. We administered steroids to control the inflammatory reaction and antibiotics to control the infection. After that, the symptoms of the children gradually relieved, and laboratory parameters of HLH were obviously improved, suggesting that the treatment was effective. During the 1-month follow-up, there was no recurrence of HLH.

For patients with recurrent infection in early childhood, we should pay close attention to the possibility of congenital immune deficiency, and genetic testing should be performed as soon as possible. Due to abnormal macrophage function in children with CGD, infection may lead to the release of a large number of cytokines, which can trigger HLH. On the basis of antiinfection, IVIG or steroids should be the first choice of HLH treatment, and strong immunosuppressive drugs, such as cyclosporine and etoposide, should be avoided.

## Data Availability

The datasets used and analyzed during the current study are available from the corresponding author on reasonable request.

## Abbreviations

CGD: Chronic granulomatous disease
CMV: Cytomegalovirus
CRP: C-reaction protein
EBV: Epstein-Barr virus
ERK: Extracellular-signal regulated kinase
FHL: Familiar HLH
GM-CSF: Granulocyte-macrophage colony-stimulating factor
HLH: Hemophagocytic lymphohistiocytosis
HSV: Herpes simplex virus
IASH: Infection-associated hemophagocytic syndrome
IFN: Interferon
IL: Interleukin
IVIG: Intravenous immunoglobulin
KC: Keratinocyte-derived chemokine
NADPH: Nicotinamide adenine dinucleotide phosphate
NF: Nuclear factor
NK: Nature killer
sCD25: Soluble CD25
TNF: Tumor necrosis factor
VP: Etoposide vepesid

## References

[1] Al-Samkari H, Berliner N (2018). Hemophagocytic lymphohistiocytosis. Annu Rev Pathol.

[2] Morimoto A, Nakazawa Y, Ishii E (2016). Hemophagocytic lymphohistiocytosis: pathogenesis, diagnosis, and management. Pediatr Int.

[3] Yanagisawa R, Nakazawa Y, Matsuda K, Yasumi T, Kanegane H, Ohga S (2019). Outcomes in children with hemophagocytic lymphohistiocytosis treated using HLH-2004 protocol in Japan. Int J Hematol.

[4] Roos D (2016). Chronic granulomatous disease. Br Med Bull.

[5] Parekh C, Hofstra T, Church JA, Coates TD (2011). Hemophagocytic lymphohistiocytosis in children with chronic granulomatous disease. Pediatr Blood Cancer.

[6] Schappi MG, Jaquet V, Belli DC, Krauze KH (2018). Hyperinflammation in chronic granulomatous disease and anti-inflammatory role of the phagocyte NADPH oxidase. Semin Immunopathol.

[7] Álvarez-Cardona A, Rodríguez-Lozano AL, Blancas-Galicia L, Rivas-Larrauri FE, Yamazaki-Nakashimada MA (2012). Intravenous immunoglobulin treatment for macrophage activation syndrome complicating chronic granulomatous disease. J Clin Immunol.

[8] Maignan M, Verdant C, Bouvet GF, Van Spall M, Berthiaume Y (2013). Undiagnosed chronic granulomatous disease, burkholderia cepacia complex pneumonia, and acquired hemophagocytic lymphohistiocytosis: a deadly association. Case Rep Pulmonol.

[9] Valentine G, Thomas TA, Nguyen T, Lai YC (2014). Chronic granulomatous disease presenting as hemophagocytic lymphohistiocytosis: a case report. Pediatrics..

[10] Jackson SH, Devadas S, Kwon J, Pinto LA, Williams MS (2014). T cells express a phagocyte-type NADPH oxidase that is activated alter T cell receptor stimulation. Nat Immunol.

[11] Marsh RA (2018). Epstein-Barr virus and Hemophagocytic Lymphohistiocytosis. Front Immunol.

[12] Han XC, Ye Q, Zhang WY, Tang YM, Xu XJ, Zhang T (2017). Cytokine profiles as novel diagnostic markers of Epstein-Barr virus-assocaited hemophacytic lymphohistiocytosis. Cytokine..

[13] Xu XJ, Tang YM, Song H, Yang SL, Xu WQ, Zhao N (2012). Diagnostic accuracy of a specific cytokine pattern in Hemophagocytic Lymphohistiocytosis in children. J Pediatr.

